# Experiences of exercise in patients with asthma: a qualitative analysis of discussions in a UK asthma online community

**DOI:** 10.3399/BJGPO.2021.0162

**Published:** 2022-08-10

**Authors:** Sara Sadek Attalla, Nadya L Ow, Melitta McNarry, Anna De Simoni

**Affiliations:** 1 Barts and The London School of Medicine and Dentistry, Queen Mary University of London, London, UK; 2 Applied Sports, Technology, Exercise and Medicine Research Centre, Asthma UK Centre for Applied Research (AUKCAR), Faculty of Science and Engineering, Swansea University, Swansey, Wales, UK; 3 Asthma UK Centre for Applied Research, (AUKCAR) Wolfson Institute of Population Health, Queen Mary University of London, London, UK

**Keywords:** asthma, exercise, strategies, primary health care

## Abstract

**Background:**

Engagement with exercise in adults with asthma is suboptimal. Limited information is available regarding factors affecting engagement with exercise.

**Aim:**

To explore experiences of exercise and linked unmet needs in adults with asthma.

**Design & setting:**

Qualitative thematic analysis of posts in a UK asthma online community, written between 2015 and 2020.

**Method:**

Posts were identified using keywords searches. Posts in the ‘Exercise’ topic section were additionally included. Thematic analysis of posts was undertaken.

**Results:**

A total of 143 relevant posts were analysed. Ninety-two participants were identified through posts (11 male, 33 female, 48 sex not stated, aged 26–73 years). Emerging themes included the following: fear of experiencing asthma symptoms during exercise; lack of information about how to deal with symptoms; external barriers; emotional response; and involvement of healthcare providers. Environmental factors, concomitant life stressors, distrust of healthcare professionals, and embarrassment about displaying asthma symptoms during exercise were barriers to engagement. Facilitators included experiencing positive health outcomes following exercise and positive discussions regarding exercise with healthcare professionals. Strategies participants developed to enable exercise were warming up, increasing reliever and preventer inhalers when exercising, and finding exercises the individual felt were enjoyable.

**Conclusion:**

Future interventions to address fears of exercise-induced physical symptoms, and clear instructions on the use of inhalers when exercising are needed. Exploring patients’ attitudes to exercise in clinical consultations, especially in primary care, may be beneficial.

## How this fits in

Exercise in patients with asthma is not routinely discussed in consultations. Patients are unsure about how to exercise and use inhalers with exercise. Receiving positive reinforcement and support by healthcare professionals is a facilitator to exercise. Experiencing exercise-related asthma symptoms triggers emotional and embarrassment responses that may be underestimated and affect subsequent engagement with exercise. Guidelines for healthcare professionals do not currently hold issue-specific instructions on management of exercise in patients with asthma. Exploring patients' attitudes to exercise in clinical consultations, especially in primary care, may be beneficial. Novel interventions aimed at raising clinicians' awareness, as well as providing practical and emotional support to patients with asthma engaging with exercise, are warranted.

## Introduction

Asthma is one of the most common chronic conditions,^1^ with 5.4 million people affected in the UK.^2^

The relationship between exercise and asthma is complex, with exercise potentially aggravating an increase in airway resistance and resulting in physical symptoms that can be offputting for patients. Exercise-induced bronchoconstriction describes a temporary narrowing of the airway that occurs during exercise.^3^ This occurs in 40%–90% of people with asthma (referred to as exercise-induced asthma) and 20% of those without asthma (simply referred to as exercise-induced bronchoconstriction).^4,5^

Exercise has shown to be beneficial, although often overlooked, in the long-term management of asthma.^6^ Previous studies assessing the impact of exercise in patients with asthma have reported positive effects on cardiopulmonary fitness, as well as improved emotional status and decreased levels of wheezing.^7^ Both the American College of Sports Medicine^8^ and American Thoracic Society^9^ recommend exercise for patients with asthma. Exercise and physical activity are important in maintenance or achievement of healthy weight status, with exercise directly impacting metabolic rate and energy expenditure.^10,11^ Obesity rates have risen substantially in the UK, with 28% of adults categorised as clinically obese based on their body mass index (BMI) in 2019.^12^ Obesity is a risk factor for the development of asthma,^13^ with the incidence of asthma increasing 2.0-fold in children and 2.3-fold in adults who are obese.^14^ In patients already diagnosed with asthma, obesity can affect both asthma control and exacerbation severity,^15^ resulting in poorer outcomes and reduced quality of life.^16^

The British Thoracic Society (BTS) states that exercise-induced asthma may indicate poor asthma control and warrants regular treatment review.^17^ Guidelines recommend the use of a short-acting beta-2 agonist (SABA) immediately before exercise as well as regular use of an inhaled corticosteroid. Where this is not sufficient to control symptoms caused by exercise, consideration of adding a leukotriene receptor antagonist, long-acting beta-2 agonist (LABA), chromones, or theophyllines is warranted.^17^

Multiple studies have reported that patients with asthma are less likely to take part in exercise, with a recent systematic review reporting 11 studies where participants with asthma engaged in less exercise than controls, versus six studies reporting no difference.^18^ Several barriers to exercise, such as fear and difficulty breathing, have been reported in the adolescent population with asthma.^19^ Lack of time is more likely to be reported as a barrier in younger patients.^20^ Fear of exacerbating symptoms is also a common theme among adolescents^19^ and adults,^20^ with patients with more severe disease more likely to view exercise as detrimental. Facilitators include the desire to be healthy and encouragement from a motivated companion or physician. In terms of intrinsic characteristics, patients with less asthma knowledge, lower self-efficacy, and more negative attitudes towards asthma are more likely to view exercise negatively.^20^ Studies examining exercise-promoting interventions in adults with asthma have focused on the involvement of more structured exercise plans and tips specifically focused on patients with asthma.^21^ Little published information was, however, found regarding strategies that patients with asthma came up with to help them exercise and the role of primary care.

Better understanding of the underlying factors that result in reduced engagement with exercise in this group of patients is needed. Online communities might include people who do not take part in traditional research studies, therefore offering perspectives from an unrepresented patient population.^22^ Such data can provide new and insightful perspectives on engagement with exercise in patients with asthma, with the potential to inform healthcare interventions.^23,24^

This study aimed to explore whether exercise was a topic of discussion in asthma online communities and to identify potential unmet needs, and barriers and facilitators to engagement with exercise.

## Method

A qualitative analysis of posts was conducted within the Asthma UK community. The Asthma UK community has more than 18 000 members and 22 000 posts.^25^ The Asthma UK forum is used by patients with asthma to share their stories, and give and receive information and support. The online community was chosen following an initial Google search, which showed a wealth of information being exchanged on exercise and asthma, as well as ‘Exercise’ being one of the discussion topics listed within the community. The authors aimed to include posts made by adults about exercising with asthma, whether discussing their own experiences and stories or providing support to others.

### Ethical issues

Ethics approval for this study was assessed by the Queen Mary’s University Research Ethics Committee and was exempt from full review. Permission was granted by both HealthUnlocked and Asthma UK before starting the study, as in previous investigations by the same authors.^26–28^ The passive analysis approach used in this study is generally considered non-intrusive.^29^ In order to protect the identity and intellectual property of forum participants, direct quotes have not been used, despite this being normal practice in qualitative research. Summative descriptions of quotes will instead be used throughout the article, as previously described.^23^

### Posts and participant identification

To identify relevant posts, the following terms were searched: 'exercise', 'fitness', 'physical activity', and 'weight', using the search facility on the Asthma UK website. Posts and threads included within the topic 'Exercise' were also included. All posts belonging to the threads were analysed, provided they were relevant to the research question. Participants were retrieved through the posts. Posts written between 2015 and December 2020 were exported into an Excel database in chronological order. To avoid third-party interpretation bias, posts written by family members or friends talking about patients with asthma were not included. Data on usernames, sex, age, asthma treatment, and asthma severity were retrieved within the posts, where available.

### Analysis

Posts were analysed using thematic analysis^30^ using a data-driven approach. SSA read all posts to familiarise with the data and participants.

Initial codes produced looked specifically at barriers, facilitators, and strategies discussed in relation to exercising with asthma on the community. NLO independently coded 20% of the posts. Disagreement was identified between the coders on three out of 30 posts, and was resolved with discussion between SSA, NLO, and ADS. Following coding, main themes and sub-themes were identified, and were iteratively reviewed and refined throughout the analysis.

Microsoft Excel (version 16.63.1) was used for data collection, coding, and statistical measures (mean, standard deviation [SD]).

## Results

Exercising in asthma was a topic of discussion, with 149 posts retrieved, 143 of which (96%, 15 701 words in total, averaging >100 words per post) were considered relevant to the research question and included in the analysis. Most posts discussed concerns regarding exercising with asthma, use of inhalers when exercising, and how to safely exercise without exacerbating asthma symptoms. Posts were written evenly throughout the years from 2015–2020 (12.2% ±8), with the exception of 2016 (39% of posts written in the year).

### Participants

A total of 92 participants were identified from 143 posts (Table 1). Most participants did not reveal personal characteristics such as sex, ethnic group, or age. No information on ethnic background or geographical location was reported. Among participants who disclosed their sex, there were three times the number of females to males (33 versus 11).

**Table 1.** Sample characteristics of the Asthma UK community participants and posts

Sample characteristics	*n*	Median (range)	Mean (SD)
**Number of unique usernames or names** ^a^	**92**		
Participant age, years (*n* = 9 posts stating age)		52 (26–73)	49.7 (15.2)
**Sex**			
Male	11		
Female	33		
Not stated	48		
**Total number of posts**	**143**		
**Number of posts mentioning asthma medication used and type**			
Preventer and reliever inhalers	39		
Reliever inhaler only	8		
Leukotriene antagonists	4		
Oral steroids	11		
Monoclonal antibody injections	2		
Not stated	79		

^a^Hidden usernames excluded.

### Themes

A range of themes relating to barriers, facilitators, and strategies to engage in exercise were highlighted in the context of exercising in asthma (see Table 2).

**Table 2.** Themes and sub-themes emerging from posts, divided into barriers, facilitators, and strategies

Barriers	Facilitators	Strategies
Experiencing physical symptoms during and after exercise Lack of information on exercising in asthma: How to participate in exercise with asthma safely How to use inhalers with exercise to prevent asthma attacks External barriers For example, cold weather and stress in private life Emotional barriers For example, fear of physical symptoms, hospital admissions, and asthma attacks Embarrassment when exhibiting asthma symptoms during exercise — confused with lack of fitness Distrust of doctors caring for their asthma Unclear medical advice about exercising	Experiencing positive psychological effects of exercise Experiencing improvement of asthma symptoms with exercise Healthcare professional involvement Regular input from a healthcare professional to adjust asthma medications Healthcare professional involvement in explaining how to exercise Positive reinforcement from a healthcare professional	Use of reliever inhalers before exercise Increase use of preventer inhalers Starting exercise slowly Warming up before exercise Finding specific exercises that one enjoys

A relationship was observed between the themes organised as barriers and facilitators, in that some users were suggesting how barriers could be overcome (for example, naming facilitators or strategies). Strategies are reported within the facilitators section.

### Barriers

#### Fear of physical symptoms

It was common for participants to report unpleasant asthma symptoms when exercising such as breathlessness, discomfort, wheeze, and burning in the chest. Post-exercise symptoms included increased phlegm, cough, and chest pain. These were considered as barriers to exercise.

A participant described that she wanted to improve her fitness levels, but whenever she attempted exercise, she experienced severe shortness of breath, a tight chest, cough and an *'itchy*' feeling in her lungs. She described that the feeling of breathlessness scared her and put her off trying to push onwards with exercising. To overcome this, her strategy involved using salbutamol before exercise, which she found helped a bit. (Female, age not stated, participant N. 7)

#### Lack of information on exercising in asthma

##### How to exercise with asthma

Participants looking for advice on how to safely start exercising with asthma wrote a number of posts. They recognised that they needed to lose weight and improve their fitness levels, and that this would help with their asthma symptoms, but they did not know how to do so without causing an asthma attack, showing a possible lack of knowledge when it comes to exercise.

A participant described that she was trying to lose weight and was very unfit but was struggling to take part in any meaningful exercise. She expressed worries that she wanted to exercise in a way that avoided her needing to take oral steroids but felt that this put her in a difficult situation. (Female, age not stated, participant N. 1)

A participant asked for advice, explaining that they really wanted to start running to lose weight but that they didn’t know how they could begin this when they already struggled with walking. They mentioned specifically wanting to avoid having an asthma attack. (Age and sex not stated, participant N. 89)

##### How to use inhalers before, during, and after exercise

Participants described their use of inhalers while exercising and suggested strategies they developed to prevent physical symptoms triggered by exercise. A common suggestion was the use of a reliever inhaler before starting exercise.

A participant explained that she was given advice from a physiotherapist to take two puffs of her reliever inhaler 30 minutes before taking part in exercise and recommended this to others on the community. (Female, age not stated, participant N. 12)

One participant described that they experienced increased phlegm while they were exercising and afterwards, and that using their Symbicort inhaler before exercise and a few times throughout the day prevented this symptom. (Age and sex not stated, participant N. 92)

### External barriers

#### Environmental factors

Cold weather and uphill conditions were considered to be barriers in many posts made by users of the community, with participants experiencing worse physical symptoms when these factors were present.

A participant described that exercise is very important to them and that they frequently run and swim, but when it is cold outside their strategy is to run indoors on a treadmill to avoid the cold air. They wrote that their asthma is worsened by the cold air and that they have to make sure they don’t run too vigorously in the spring or autumn so that they don’t have a bad asthma attack. (Male, age not stated, participant N. 73)

#### Stress from work and private life

Stressful life circumstances were also reported as a barrier to exercise and to affect ability to take part in exercise. The most common stressors mentioned were family worries and occupational concerns.

A participant described that he believed the biggest factor in the development of his asthma was stress from his work and his personal life. Before this he could run long distances a few times a week, but now required medication to be able to do so. He also described that he has young children, one with a severe disability, which has also been difficult for his family and him to cope with. (Male, age mid-40s, participant N. 6)

Another participant described that she has had to reduce the amount of exercise she takes part in as she had experienced increasing asthma flare-ups in the last few years. She described that stress is a big trigger for her and she found this very hard to avoid as she has multiple children who have asthma and one child with a developmental disorder. (Female, age not stated, participant N. 21)

### Emotional response

#### Fear of negative symptoms or outcomes

Participants described fear of worsening their asthma with exercise and potentially experiencing negative physical symptoms, asthma attacks, or hospital admission. Negative emotional symptoms were also reported following exercise, including embarrassment and shame.

A participant wrote that they visited their asthma nurse who explained to them that stopping exercise would have a negative effect on their asthma, however they were too scared to continue with exercise after previously having to visit A&E following an asthma attack triggered by exercise. (Age and sex not reported, participant N. 28)

A participant wrote that he used to run long distances a few times a week, but now could only do this following a course of steroid tablets and in warm weather conditions. He used to take part in kickboxing and tried to return to this recently, but really struggled at his class and felt ashamed of his fitness levels. He thought that his instructor looked very worried about him due to his breathlessness, and after his experience did not return to this class. (Male, age mid-40s, participant N. 6)

### Facilitators

#### Positive effects of exercise

A positive impact on asthma symptoms, fitness levels, and mood encouraged exercise uptake in asthma and persistence with exercise. This was also reported by older users of the community.

One participant wrote that following a hospital admission their peak flow was lower than previously. They began to run with their dogs three times a week and found that their peak flow had increased substantially. This made them feel positive about their next appointment for their asthma check-up. They described feeling a good improvement in their lungs since they started to exercise more and that they were in a better mood. (Male, age 68, participant N. 80)

Another participant described that they felt better both physically and mentally when they take part in regular exercise. They explained that this made them feel that they had a level of control over their health and asthma, a feeling that they described was sometimes lost when their asthma symptoms were flaring. (Age and sex not reported, participant N. 60)

#### Involvement of healthcare professionals

##### Regular asthma medication monitoring

Regular asthma medication monitoring was shown to have a positive impact on uptake of exercise in patients with asthma. Being able to discuss any asthma symptoms experienced in relation to exercise with a trusted healthcare professional allowed better adjustment of participants’ medications and encouraged them to continue exercising.

A participant described that she was struggling with soreness in her chest and that any form of exercise was causing her asthma symptoms to worsen. She wrote that she saw her GP and was advised to increase her Ventolin dose, with four puffs to be taken before exercise. Since increasing this, she had been able to do a lot more exercise and encouraged other posters to raise issues with their asthma nurse and GP. (Female, age not reported, participant N. 34)

##### Relationship with healthcare professional

The relationship between the participants and their healthcare providers had an impact on people’s engagement with exercise. Positive reinforcement and encouragement given by asthma nurses and doctors to continue with exercise was recognised as a facilitator to exercise in participants on the community, whereas a lack of trust in the medical advice acted as a barrier to engagement with exercise.

A participant wrote that they discussed their exercise with their asthma consultant who was happy with their exercise levels and encouraged them to persevere. The participant wrote that it took them longer to reach their exercise goals, but that they could now run long distances every few days and have to use their inhaler less frequently than they did before. They encouraged others on the community to keep going and explained that they felt it helped both their physical and mental health. (Age and sex not reported, participant N. 35)

A participant wrote that she was worried about making an appointment with her asthma nurse or GP as she felt that they were both *'rubbish*'. She described that she felt her asthma nurse had given her incorrect advice in relation to her peak flow and told her that her asthma could not be that bad if she was not wheezing. She felt that because her asthma symptoms were less obvious, she was dismissed by her nurse and GP who may think it was *'all in her head*'. (Female, age not reported, participant N. 7)

Another participant described that their doctor wanted them to begin a '*graded exercise programme'* to rebuild strength and prevent relapses in their asthma. They described that the doctor encouraged them to do a bit more each day, and that they felt this was too vague and they didn’t know what to do. They wrote about being concerned that they would overdo the exercise and could become very unwell again. (Age and sex not reported — hidden username group).

### Use of medications

Issues around asthma medications and exercise included participants being unsure about using their inhalers as much as they needed during exercise, or whether there was a limit to this in terms of safety. Participants also reported that they were worried that needing to increase their inhalers during exercise could indicate that they had exercised too hard and negatively affect their lungs. There was confusion as to whether they should increase their preventer or reliever inhalers as a precaution before exercise and which would be more beneficial. Some participants reported that they were unsure as to whether they needed to increase their inhaler use or change the type of asthma medication, or whether this was a normal thing to expect because of their asthma. Some participants described that they could not exercise without first taking a course of oral steroids because this helped with their exercise tolerance.

## Discussion

### Summary

Engagement with exercise in asthma is a topic of discussion in online asthma communities, with participants revealing unmet needs, barriers, and facilitators. The data suggest that exercise in asthma is not routinely discussed in primary care consultations and patients are unsure about how to exercise and use inhalers with it. Receiving positive reinforcement and support from healthcare professionals was a facilitator to exercise. Encouragement to persist with exercise leads to better outcomes in terms of engagement with exercise, positive mental health benefits, and reduced long-term symptoms. Experiencing exercise-linked asthma symptoms also triggers an emotional response, which may affect subsequent engagement.

### Strengths and limitations

A main strength of this work was the use of an established online community.^21^ Results were based on participants’ agendas and allowed views to be identified that may not have been captured otherwise,^22,31^ from a wide geographical location. Online discussions are self-initiated and people communicate with each other without time, length, or behavioural constraints, offering a window to understand patients' issues that bypasses reactivity and self-representation bias of traditional research approaches. Additionally, online communities might include people who do not take part in traditional research studies, therefore offering perspectives from an unrepresented patient population.^22,31^ Despite the inability of the authors to ask clarification questions, users could read and reply to each other’s posts in an asynchronous way, contributing to the topic on an ad hoc basis in their own time in a single post. Participants’ insights on topics were offered without the need of facilitation and multiple interactions, unlike during face-to-face interviews or focus groups. As such, online discussions could even enable a more in-depth exploration of themes compared with interviews.^30^

The main limitation was the lack of information about participant characteristics in terms of age, sex, asthma medication, and socioeconomic factors. This made it difficult to recognise patterns within groups of people and potential common features that may have played a part in their experiences with asthma and exercise.

There is an element of participant selection and sample bias, as only the views of those taking part in the Asthma UK online community were represented. The number of participants who wrote 7% of the posts could not be determined, as 10 participants used a ‘hidden’ username. The authors cannot be certain that each username represented a distinct participant, as members can create multiple accounts. Community participants were required to be aged >16 years, meaning that younger adolescents could not be included in this study who may have had different experiences with exercise than adults.

### Comparison with existing literature

The results of this study align with the barriers to exercise seen in previous literature.^20,32,33^ These studies report that despite patients with asthma recognising the importance of exercise, they may not participate owing to barriers such as cold weather, lack of motivation, and perceived symptom burden.^32^ In one of these studies, it was found that those with more severe asthma were more likely to hold beliefs that exercise was not good for them, and that patients with worse attitudes and less knowledge about their asthma were more likely to have negative ideas about exercise.^20^ Other barriers, such as fear of worsening asthma symptoms, feeling unsafe to exercise, and exacerbation of asthma, were also described in previous literature.^20^ Facilitators to engagement with exercise were identified such as improved psychological wellbeing, asthma control, and encouragement to exercise from healthcare professionals. These mirror what has been previously described in literature.^20^

There is limited research assessing the psychological barriers to exercise in asthma. The results of the present study found that the most common barriers included fear of breathlessness and unpleasant symptoms, embarrassment owing to lack of fitness, and anxiety about overall worsening of their asthma. These barriers represent key targets for future interventions seeking to improve engagement with exercise in asthma. However, there are only a small number of studies exploring intervention development, and, in many studies, the relationship between exercise and the emotional aspects patients experience is lacking. Indeed, while Clarke and Mansur^33^ called for more research investigating the effect that fear of exercise has on asthma worsening,^33^ there remain limited new data.

Few studies have explored the effect of exercise interventions on asthma improvement and uptake of exercise. Freitas *et al* ^34^ carried out a randomised controlled trial where one group was given a behavioural intervention consisting of education and physical activity counselling.^34^ Participants in the intervention group had improved asthma control, physical activity levels, sleep quality, reduced sedentary time, and reduced anxiety symptoms compared with controls.^34^ One of the main barriers was lack of information, despite the willingness to take part in exercise.

Within the present study, specific characteristics between the participants that may have impacted their experiences with exercise could not be determined. Previous studies have aimed to assess this and found that several factors including BMI and age of diagnosis may be significant. Those with higher BMI perceived higher barriers to exercise than those with lower BMI, similarly to those who were diagnosed with asthma at an older age compared with those diagnosed before age 5 years.^33^

### Implications for research and practice

Awareness of the significant barriers to exercise, which are most important and commonly experienced by patients, may lead to improved clinical consultations with better outcomes and compliance with exercise. National Institute for Health and Care Excellence (NICE) guidelines for healthcare professionals regarding engagement with exercise in other respiratory conditions, such as cystic fibrosis,^35^ indicate that an individualised exercise programme that considers patients’ abilities and preferences should be formulated and reviewed regularly.^36^ Similarly, in chronic obstructive pulmonary disorder (COPD), the guidelines recommend that pulmonary rehabilitation programmes should be available to all patients diagnosed with COPD.^37^ Similar guidelines should be developed for clinicians treating patients with asthma. Specific instructions on the use of inhalers with exercise are required and should be discussed with patients, especially in primary care.

Discussion regarding exercise in clinical consultations is a facilitator to engagement with exercise. This allows identification of barriers such as fear of worsening asthma and physical symptoms, and addressing the lack of knowledge surrounding how to use asthma inhalers safely while exercising. The data suggest that this is not routinely happening and call for increasing clinicians’ awareness, exploration of issues with exercise, and addressing them during consultations. Formulating realistic plans and having open discussions regarding concerns is likely to be beneficial. Indeed, participants with little understanding, or who were given vague recommendations on exercise, were less likely to feel confident when taking part in exercise.

Attention to the emotional barriers to exercise (such as fear of or embarrassment when displaying asthma symptoms during exercise) is also warranted, as these are not currently addressed within guidelines.^17^ There is a need to explore the impact of mood and interoception (the sense of the internal state of the body) on (fear of) symptoms of asthma,^36^ with a specific focus on exercise.

Novel interventions aimed at raising clinicians’ awareness, as well as providing practical and emotional support to patients with asthma engaging with exercise, are warranted.

## Data

In order to protect the identity and intellectual property of forum participants, direct quotes have not been used in the article nor would it be appropriate for these to be made available by request to the authors.
